# Impact Propagation in Airport Systems

**DOI:** 10.1007/978-3-030-69781-5_13

**Published:** 2021-01-28

**Authors:** Corinna Köpke, Kushal Srivastava, Louis König, Natalie Miller, Mirjam Fehling-Kaschek, Kelly Burke, Matteo Mangini, Isabel Praça, Alda Canito, Olga Carvalho, Filipe Apolinário, Nelson Escravana, Nils Carstengerdes, Tim Stelkens-Kobsch

**Affiliations:** 8 Norwegian Computing Center, Oslo, Norway; 9 University of Trento and Fondazione Bruno Kessler, Trento, Italy; 10 Università degli Studi di Genova, Genoa, Italy; 11 IEIIT Institute, Consiglio Nazionale delle Ricerche (CNR), Genoa, Italy; 12 SINTEF A.S., Oslo, Norway; 13 Engineering Ingegneria Informatica S.p.A., Rome, Italy; 14 Instituto Superior de Engenharia do Porto, Porto, Portugal; 15 University of Padua, Padua, Italy; 16 Fraunhofer Institute for High-Speed Dynamics, Ernst-Mach-Institut, EMI, Am Klingelberg 1, 79588 Efringen-Kirchen, Germany; 17 DGS S.p.A. - NIS Network Integration and Solutions S.r.l., Via XX Settembre 41, Genova, 16121 Italy; 18 GECAD - Research Group on Intelligent Engineering and Computing for Advanced Innovation and Development, School of Engineering (ISEP), Polytechnic of Porto (IPP), R. Dr. António Bernardino de Almeida, 431, Porto, Portugal; 19 INOV-INESC INOVAÇÃO, Rua Alves Redol, nr. 9, 1000-029 Lisboa, Portugal; 20 Institute of Flight Guidance, German Aerospace Center (DLR), Braunschweig, Germany

**Keywords:** Airport, Cyber-physical attack, Impact propagation

## Abstract

The effective protection of critical infrastructure against cyber and physical security threats involves many different steps from initially the identification of risks to finally the implementation of counter measures in the infrastructure. To derive counter measures and to come to intelligent decisions facing the identified risks, the impact calculation plays a central role. The impact of a specific threat can propagate through the systems of the infrastructure and thus needs to be analysed carefully. In this paper, the role of impact propagation of cyber-physical threats for infrastructure protection is discussed, exemplified for airport systems. In the ongoing EU-H2020 project SATIE (Security of Air Transport Infrastructure of Europe) a toolkit is developed containing two tools for impact propagation, namely the Business Impact Assessment (BIA) and the Impact Propagation Simulation (IPS). Both tools are described and for a small test case the propagation of a cyber threat and the transformation into a physical threat is demonstrated in a network representation as well as an agent-based model of the airport’s systems employing the IPS.

## Introduction

In the world of critical infrastructure, the systems involved have direct and indirect interdependencies and are vulnerable to each others threats, risks, impacts and disruptions, whether deliberate or accidental, [34]. Interdependencies between systems are crucial for the protection of critical infrastructures because they can allow a failure which is seemingly isolated in one system to cascade and impact several other systems [39]. For example, when there are severe weather conditions at one airport, turning down its throughput, this causes delays and can cause a whole continent to feel the effects as the delays propagate to all other connected airports, creating further delays as the aircrafts continue their flight schedule to other destinations, but with the delays ever more compounded [30]. This cascading effect is predictable with appropriate propagation models.

Cyber-threats are by no means immune to this phenomenon as they can quite easily spread across networks, especially in our increasingly connected world, but also cyber-threats can even turn into physical or even safety threats [17]. Cyber-threat impact assessment allows organizations to understand how a cyber-threat can damage their information technology (IT) infrastructure and identify the critical services that could be impacted by the threat. These techniques distinguish themselves in three key points: impact modelling, propagation and assessment.

For impact modeling, a correlation model is often constructed using an interconnected graph [1, 7, 11, 12, 21, 28, 29, 31, 43] that profiles relationships between organization assets and critical services. Some portray impact modelling [1] by inspecting communication exchanged by IT devices to represent the organization’s environment as an interconnected graph. Other works [12] expand this graph to also include a higher-level mapping between assets and the organization critical services. Also, some studies [43] integrate a security layer to include information about how vulnerable assets can influence impact propagation.

Within the air transport environment, it is not difficult to imagine how risks to a cyber-attack can result in potential physical damage. In fact, a ransomware attack to a Cleveland Airport server in April of 2019 prevented the displaying of the flight information to passengers, preventing them from finding the correct gate, causing confusion, crowds, and panic among the passengers, then overburdening the airport staff, and causing flights to be grounded [27]. There was no further physical attack, but the risks to one were greatly increased because of that single cyber-attack.

Airports in particular are highly complex systems with a multitude of stakeholders, each of them with their own intentions and goals. Various interconnections exist between these stakeholders, and between the assets of the airports (e.g. airports themselves, baggage, passengers, etc.), making it difficult to assess the impact of a particular action on the whole airport and even system of connected airports. Therefore, it is vital to quickly detect threats and understand its potential impact on the rest of the complex system. This is not a novel issue, though, so there have been some attempts at aiding this understanding of propagating threats and coordinating efforts between stakeholders. The first was the airport collaborate decision making (A-CDM, [5, 6]), which just shared some basic airside information (e.g. the Target Off-Block Time, TOBT). Then the Total Airport Management (TAM, [9, 36]) research aimed to provide stakeholders with a holistic view of the airport, its performance and causes for degradations. The necessity of better collaboration was underpinned by a job and task analysis at German airports which revealed that coordination was still lacking between stakeholders, and airport performance was sub-optimal due to the lack of timely or precise information [35]. As a concept, TAM is built around the idea of an Airport Operations Centre (APOC) where the main stakeholders are collaboratively working on a plan to improve the airport performance. More recently, [41] presented a concept for a what-if tool for TAM, to support decision-making and predicting overall effects of stakeholder actions and the comparison between several alternatives.

The lessons learned in TAM can be transferred to security research in aviation as shown by SATIE. Here it is foreseen that security practitioners and airport managers collaborate more efficiently during a crisis to achieve its mitigation. This can be done in a security operation centre (SOC), where the operators are informed about alarms and the reasons of the alerts. In a SOC emergency, procedures can be triggered simultaneously through an alerting system in order to reschedule airside/landside operations, notify first responders, cyber-security and maintenance teams towards a fast recovery. Information about impact propagation information will ultimately improve the situational awareness of the decision makers and will lead to better and faster decisions.

However, the availability of additional information may also overwhelm human operators and, especially in these situations, an automated decision support has the potential to assist the quick resolution of a critical situation or even a crisis [2]. Therefore, a unified tool, taking into consideration all of the physical and digital assets involved, their interconnections, and how threats can propagate through the system would be ideal.

The mechanisms of probabilistic propagation between assets is applicable in a variety of fields, but is particularly critical in the air transport world where lives are at stake. Effective prediction of such effects allow for much more efficient and responsive countermeasures to reduce those risks at a single point which could become devastating once they propagate through the system. In this paper, impact propagation in airport infrastructure and the corresponding systems is discussed in the context of the project SATIE. Two systems of the SATIE toolkit perform this type of impact analysis, namely the Business Impact Assessment (BIA) and the Impact Propagation Simulation (IPS), which are described in Sect. 2. In Sect. 3, the IPS is applied to a small test scenario where cyber-physical threats impact some specific airport systems. Note, we present preliminary results of the tool development in the course of the ongoing project. In Sect. 4 the results are summarized and an outlook is given.

**Fig. 1.**
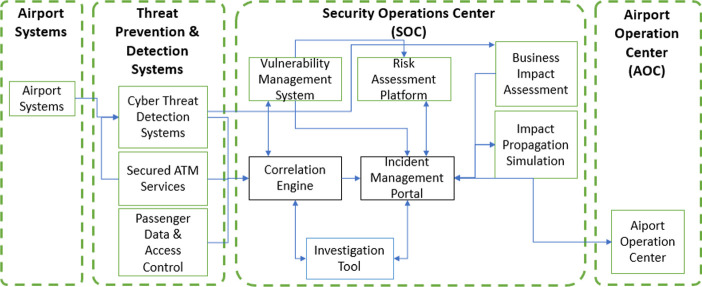
Simplified architecture of the SATIE toolkit.

## Approach

### SATIE Toolkit and Ontology

SATIE proposes an architecture that combines new and existing tools in order to timely detect cyber-physical incidents, simulate their impact and deliver information to the Security and Airport Operation Centers. Live data is captured through several sensors and existing tools in the airport systems, which is then analysed through several threat prevention and detection systems. These systems will analyse data according to different perspectives and may describe events and trigger alarms. These are sent to the Security Operation Center, namely to the Correlation Engine, which will attempt to find possible correlations between the messages obtained through the different sources. Querying either the Vulnerability Management System and the Risk Assessment Platform will supply additional information about the events that generated the alerts, particularly regarding possible vulnerability exploitation and affected assets. Information is centralized in the Incident Management Portal, where a human operator can opt to aggregate different events into incidents and query the impact simulation tools in order to know which assets could be affected by a single incident, and obtain possible mitigation strategies. Figure 1 shows a simplified version of the overall SATIE architecture. The IPS receives incidents from the Incident Management Portal and requests for simulation. It facilitates to the Incident Management Portal a visualization of possible threat propagation paths and mitigation strategies, depending on the threat under consideration.

An ontology is developed in SATIE to describe the contexts of the exchanged messages, namely the sub-domain of incidents, impacts and assessments. The impact’s specification is directly related to the needs of the BIA and IPS, describing how the performance of assets may be affected, how assets affect each other and suggesting possible mitigation strategies, each with their own expected performances. How different events and assets may affect each other is described by the threat propagation path and the threat propagation event concepts respectively.

**Fig. 2.**
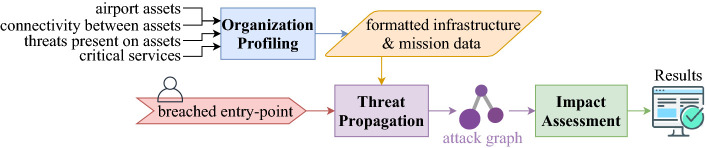
Architecture of the Business Impact Assessment (BIA)

### Business Impact Assessment (BIA)

The SATIE toolkit includes the BIA, that analyses how cyber-threats propagate to the organization assets and assesses the impact caused on the organization’s business critical services and goals. As can be seen in Fig. 2, BIA performs impact assessment in three stages, organization profiling stage, threat propagation stage and impact assessment stage. At the first stage, BIA gathers information about the organization’s infrastructure using reconnaissance techniques [22] that automatically query inventory system and continuously inspect IT network traffic to discover the organization assets, and how threats can propagate by means of communications between organization assets. The system also resorts to process mining techniques [45] for identifying asset involvement in the organization critical services by inspecting registry of operations performed by each asset. At the second stage, BIA simulates the propagation of a user-chosen threat based on an attack graph model, where the goal is to determine whether a compromised asset is likely to deleteriously affect any of the critical services of the organization. To this end, logic programming and attack graphs are used to express the rules and preconditions that must be met for threat propagation to occur [33]. At the third stage BIA traverses the attack graph to identify assets affected by threat propagation and determine the critical services compromised. The result of this final analysis produces a report of the cascading effects the simulated threat had on the organization infrastructure, highlighting the assets affected in each threat propagation steps and critical services potentially damaged by the threat. Ultimately, this final report may be used to aid risk analysis by simulating impact propagation of a potential exploited threat.

For impact propagation, a model-based analysis is used and can be categorized into *logic-based models* [1, 3, 4, 12, 14, 18, 21, 28, 29], *probabilistic-based models* [3, 23, 37, 42] and *sensitivity-based models* [7, 20, 24–26, 31]. Logic-based analysis approaches are based on an attack graph model that gradually analyzes the cascading effects a cyber-threat can have on the organization assets and how they can be exploited to compromise the critical services. Probabilistic-based impact propagation often uses Bayesian Networks to express how assets can be compromised and how critical services are impacted by conditional probabilities. Sensitivity-based approaches use active perturbation by purposely compromising an asset to identify how it impacts the critical services. For impact assessment, several works distinguish themselves on how they evaluate the impact propagation cascading effects on asset and critical services operability. Some use qualitative metrics [8, 15, 16, 21, 37, 38], to evaluate the overall impact based on risk categorization on how vulnerable assets and critical services are to cyber-threats and how easily they can be exploited by attackers. Other works use quantitative metrics [12, 14, 16, 43], to measure how cyber-threats impacts the operationality, exposure and efficiency of assets and critical services.

### Impact Propagation Simulation (IPS)

The IPS follows a different approach than the BIA, i.e. the focus is more on system’s assets and passenger behaviour than on business processes. Furthermore, the IPS accounts for cyber and also physical threats. Even if this is not foreseen in the project, the BIA and IPS approaches could be integrated in future work. The IPS is based on two complementary modeling approaches, namely a network and an agent-based model.

**Network Model.** The network model of IPS is based on CaESAR (Cascading Effects Simulation in urban Areas to assess and increase Resilience), a tool developed at Fraunhofer EMI [10]. This tool simulates networks topologically as nodes and edges. In the context of airports, the nodes are assets such as serves, card readers, switches and edges are e.g. cables, information exchange or influence in case of an incident. Using node and edge lists, more details can be implemented into the simulation, including the direction of flow with the edges, and the mean time to repair for the nodes.

Different types of threats can be modelled including ones that only attacks certain types of nodes, or threats that change or move over time (such as a natural disaster), or ones that only affect nodes in a specific region. Once an adverse event is simulated, nodes are removed from the model as they are damaged. Once the threat has completed its propagation, the recovery begins with the nodes being added back to their network once the repair times have finished.

Mitigation measures can also be implemented into this simulation tool which allows for resilience improvements to be made. Different measures are selected and the simulations are completed again. The effectiveness of the measures are compared utilizing the performance time curves that the tool outputs. The measures can have different effects on the different aspects of the curve, and thus effect the system’s resilience. Effects can be seen for example, on the damage sustained, or the recovery required. Depending on the goal for the resilience of the networks, the best mitigation measures can be determined. Additional models can be combined in CaESAR to determine how the threat can propagate to other networks and investigate cascading effects. This can be done with very different networks such as e.g. telecommunications, power and water networks.

**Fig. 3.**
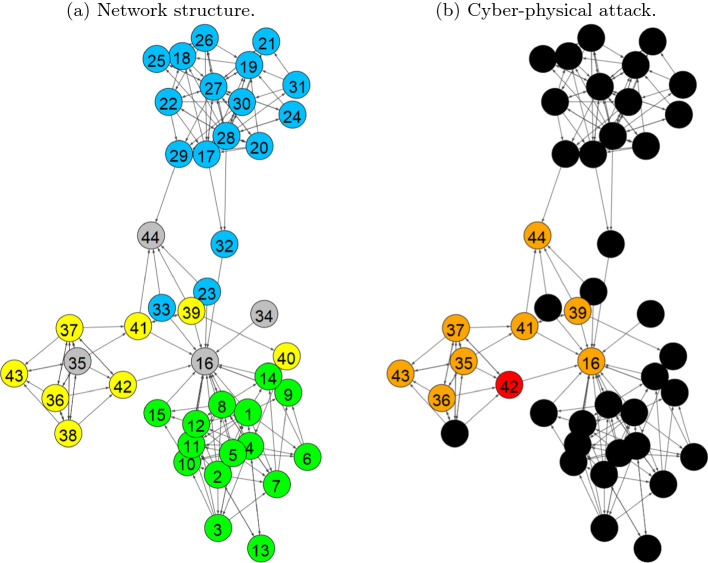
Network structure for test scenario, involved systems are: Physical Access Control System (green), Flight Information Display System (FIDS) (blue), Public Announcement (PA) System (yellow) and others (grey). During the cyber-attack: The initial attack is placed on node #42 (red) and propagates to #43 finally impacting #44 and #16. The impacted nodes and the nodes of the propagation path are given in orange. (Color figure online)

**Agent-Based Model (ABM).** To observe an infrastructure in various threat situations and to explore possible states of the system, ABM can be employed [44]. ABM has been applied in different fields such as e.g. train logistics [32], air traffic management [40] and evacuation in case of fire [13] and respective languages and platforms have been developed [19, 46]. In this paper, we present an ABM approach especially developed in SATIE to model and simulate the behaviour of passengers in an airport. Passengers are modelled as individual agents that move independently in the airport dependent on their current situation and state. The path in the airport depends on passenger attributes such as number of bags to check-in, online check-in, walking speed, memory, flight number and airport attributes such as treatment times and general layout. The walking speed, number of bags and online check-in vary randomly. The flight number decides on the check-in desk and gate. The memory of each agent influences how often they check the Flight Information Display System (FIDS) monitors to get information on their flights. Further, they follow public announcements (PA) and will receive new goals to walk to as announced. For both, FIDS and PA it is assumed that passengers follow the requests announced or displayed without questioning. However, more aspects of human behaviour will be taken into account in the further development of the implementation.

**Hybrid Model.** The transformation of threats from cyber to physical motivated the creation of a combined model. In the project SATIE, a hybrid model consisting of a network representation and an ABM is developed. The network threat propagation is triggered by received incidents, as described in Sect. 2.1 and the ABM is executed once a transformation from cyber to physical is detected or critical nodes such as employees or passengers are endangered. The network model enables the holistic overall view and the ABM gives insight into detailed processes and passenger behaviour in the airport infrastructure. The goal of the hybrid model is to combine the holistic and detailed view to enable impact propagation on each level of detail whenever it is required.

## Application

### The Test Scenario

The airport network structure for impact propagation is based on three airport systems, namely the Physical Access Control System, the FIDS and the PA System. The corresponding network representation is give in Fig. 3(a). These networks are interrelated and the main connecting nodes are #16 and #44, which represent employees and passengers respectively. This finding underlines the importance of the ABM. A detailed representation of passengers and employees in the infrastructure enables to better understand the interrelations between airport systems. The test scenario that is employed in this paper to test and demonstrate the impact propagation using the hybrid model is based on a cyber-attack on the PA system. During normal operation and without any threat or interruption, the attacker announces an evacuation of the whole terminal. Here, we simulate (i) the propagation of the cyber-attack in the network and (ii) the impact of the evacuation announcement on the passengers with the ABM.

### IPS Results Based on the Network Model

The scenario assumes an attack on the workstation, node #42, which is linked to the remote PA server via connecting components #37, #36, #35 (in this order). The PA server holds the database (DB) of pre-recorded messages to be used for announcements in the airport. Another node, the remote station, node #41, directly depends on the server and is also connected to it in the same manner (see Fig. 3(b)). The scenario is divided into two simulations, i.e. (i) when no mitigation option is applied during the scenario and (ii) when the PA server has installed a software for mitigation which identifies unauthorized requests for modification in the DB.

**Fig. 4.**
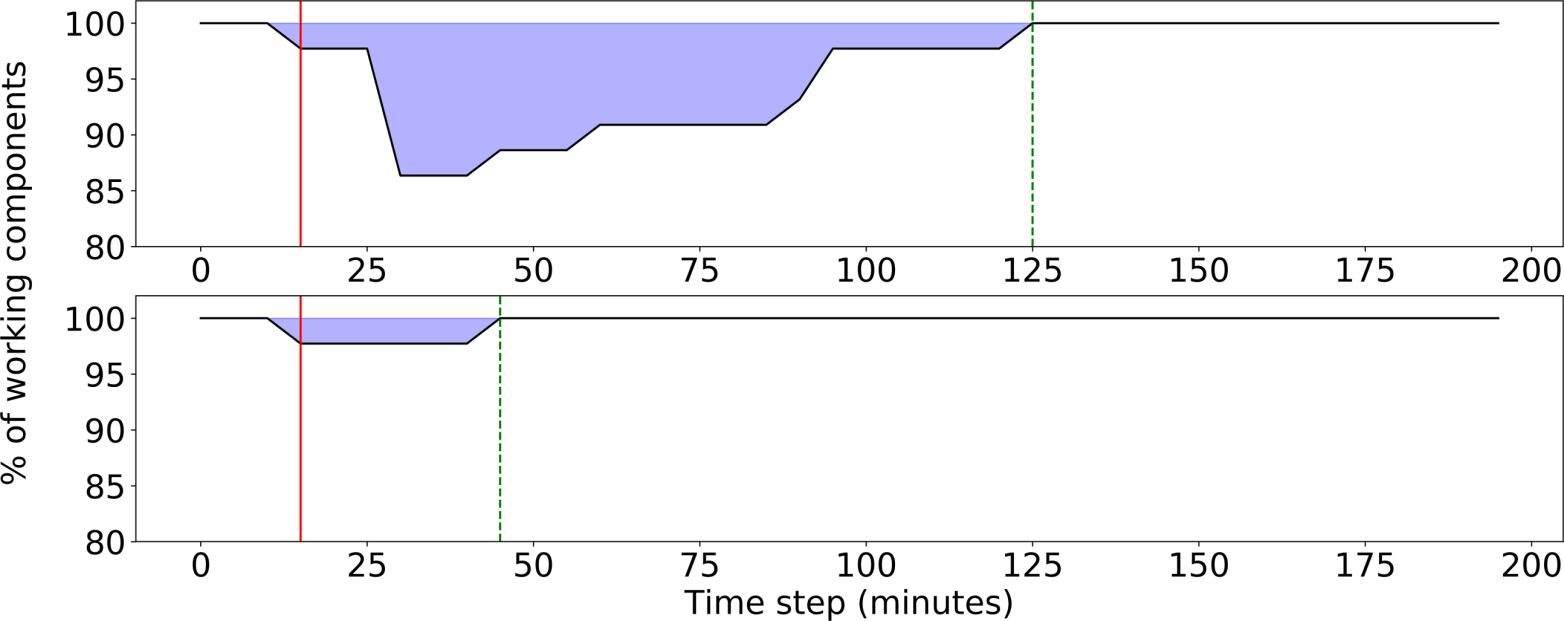
Performance of the network during the test scenario. The vertical solid red lines shows the impact on the workstation and the vertical dashed green line shows the full recovery. The blue area above the curve represents the impact and the system’s resilience. **Top**: No mitigation option. **Bottom**: With mitigation option. (Color figure online)

In absence of this mitigation measure, the workstation is attacked at $t = 15$ mins in the simulation. This is shown in the upper graphic in Fig. 4 as the red vertical line. With the access to the workstation, the server credentials are stolen and a faulty entry is updated in the DB. The attack on the DB is placed in the simulation at $t = 30$ mins. As a result, all the connected components and the PA amplifiers (node #39) are assumed to be compromised and as an impact the employees and the passengers are supposed to evacuate the airport building. It is assumed that it takes 30 min to identify and clear the workstation of the malware and another 30 min to restore the server to the previous working stage. As soon as the server is restored, all the connected components are considered to be working as expected in the next time step (5 min later). The passengers take another 35 min from this stage to get to their normal operating stage (represented by the green vertical line in Fig. 4).

In presence of the mitigation technique, the workstation is attacked at $t = 15$ mins, but the server is not impacted. So, all the connected components are shielded from the attack and the airport operates in normal manner, but with reduced performance, as the workstation is not functioning properly (see the lower graphic in Fig. 4). It takes 30 min to restore the workstation and the performance of the system is back to 100% at $t = 45$ mins (green vertical line). These results are not very surprising but demonstrate the principal that applies in small networks with limited mitigation possibilities. The impact propagation and mitigation gets easily quite complex in larger networks which will be considered in the course of the project.

### IPS Results Based on the ABM

**Fig. 5.**
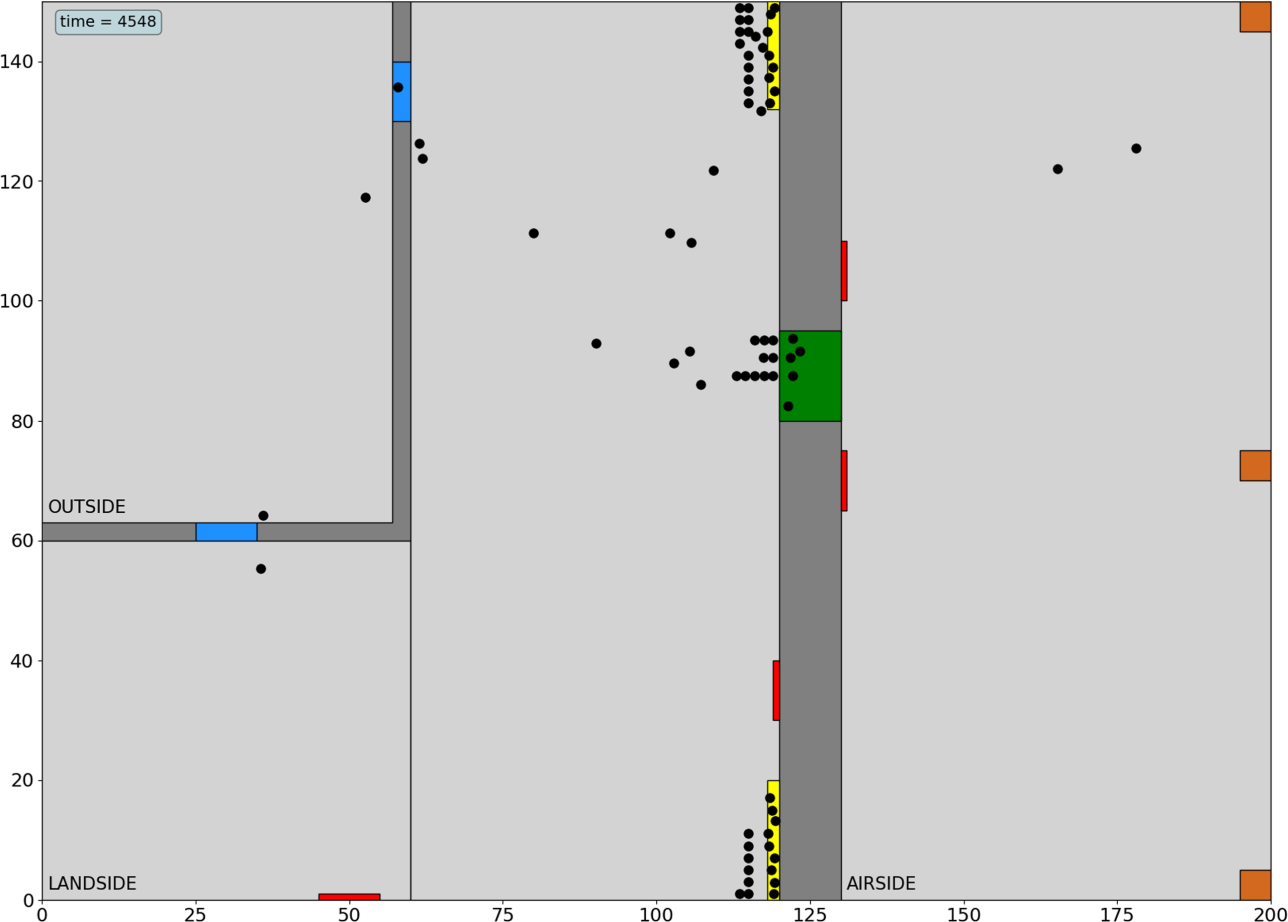
Layout for the ABM containing doors (blue), check-in areas (yellow), security screening area (green), gates (brown) and FIDS monitors (red). Agents are presented as black dots. (Color figure online)

Once the server and the adjacent nodes are compromised, it is assumed that the PA system can be manipulated. In this scenario, the evacuation of all passengers is announced which is represented in the network model by an effect on asset #44. However, the degradation and restoration of this node in the network can only be roughly assumed. At this point the hybrid model triggers the ABM to better understand and estimate the impact on the passengers. First, normal operation is simulated with the ABM, which is passengers entering the airport from the outside area, going to check-in and bag-drop in the landside area and moving though security to the airside area and gates. In the simulation, passengers are assumed to move from the gates directly to their planes. The airport layout and simulation under normal conditions is presented in Fig. 5.

**Fig. 6.**
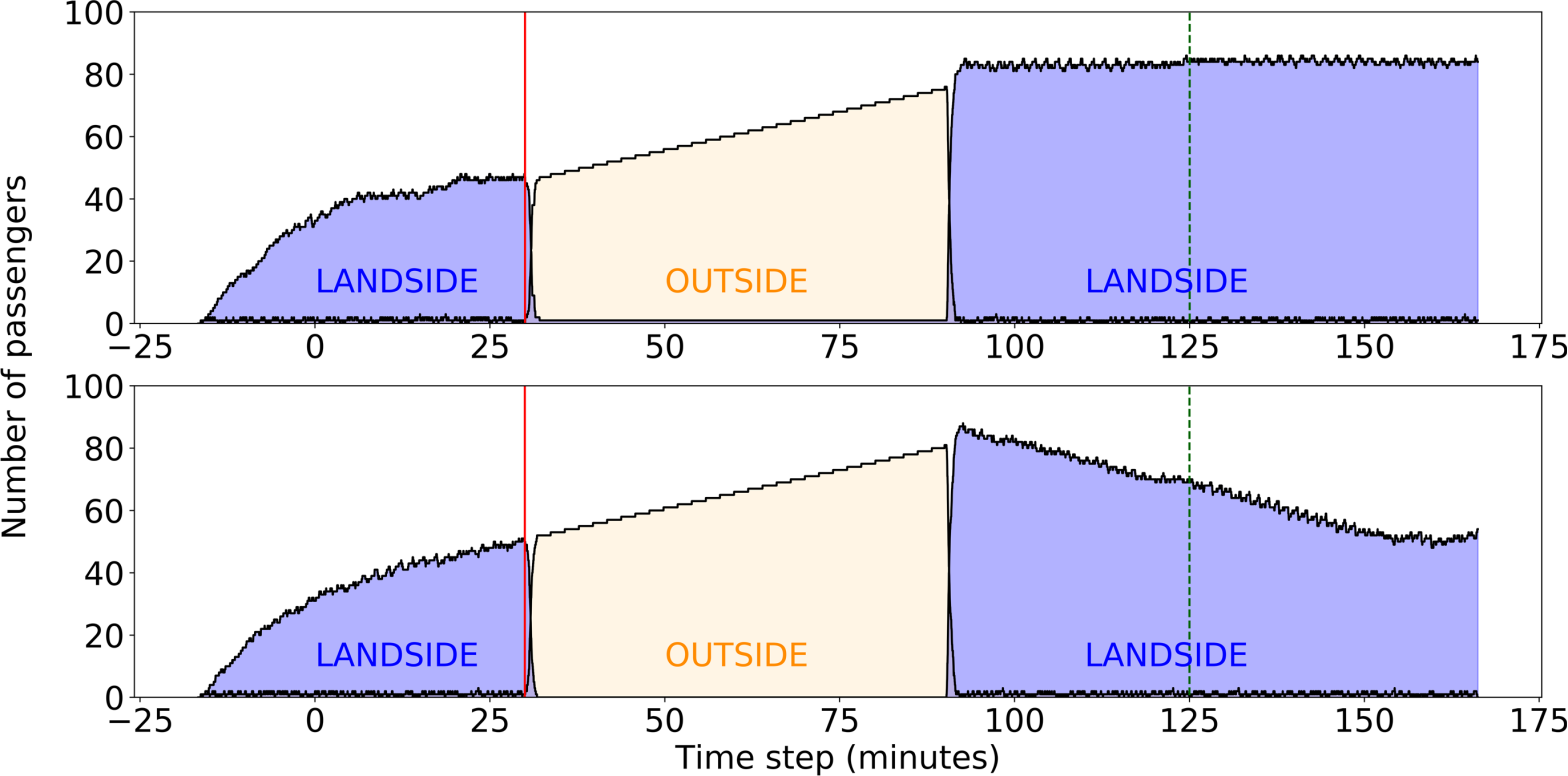
Passenger behaviour in two of the airport areas, i.e. outside (parking area) and landside as a function of time during the test scenario. The area below the curve for passengers on the landside is given in blue (dark area) and for passengers on the outside in yellow (bright area). At $t = 30$ mins the evacuation is announced (vertical solid red line). At $t = 90$ mins the passengers are allowed to enter the airport again. For reference the time the network has fully restored after the attack without mitigation option is given as vertical dashed green line at $t = 125$ mins. **Top**: No mitigation option. **Bottom**: With mitigation option: The time for passing the security check was reduced assuming more desks have been opened. (Color figure online)

Second, at $t = 30$ mins during the simulation (vertical red line in Fig. 4), the evacuation is announced. Passengers in the airside area, will leave the airport towards the gates. All other passengers leave the airport towards the outside or parking area. The respective number of passengers in each area are shown in Fig. 6.

Note, that the number of passengers in the airside area is not given in Fig. 6 for readability of the graphic. From minute $t = 10$ on passengers arrive at the airside and converge to a relatively constant number of around 5 passengers. During the evacuation they leave to the gates and after the reopening of the airport the number rises again to around 5 passengers. Further, the time of the simulation starts at $t = -16$ mins representing a correcting factor to align the time of the announcement of the evacuation between the network model and the ABM at $t = 30$ mins. In the ABM the evacuation was triggered 16 min later than in the network model as it needs some time to converge. This needs to be taken into account when further developing the hybrid model.

It is shown in the upper plot of Fig. 6 that the original number of passengers before the attack happened, is no longer reached because a crowd of people is entering the airport after the evacuation and additional passengers arrive at the airport. It is observed that the current layout of the airport and the respective number of security check desks is not sufficient to handle the large number of passengers. Therefore, a mitigation option has been introduced (see Fig. 6, lower plot). The treatment time at security check desks is reduced which is assumed to be equivalent to opening additional desks. The number of passengers after the evacuation again increases but the additional capacities at the security check helps to normalize the number of passengers landside at around $t = 150$ min.

Finally, we observe for the small airport layout and the basic principals in use that attack situations impact the airport processes and that normal operation is interrupted. The test scenario shows how the passengers gather in the outside area which represents the transformation of the attack and the corresponding impact from cyber to physical. Further, the crowd of passengers forming in the parking area might be an easy target for additional physical attacks. In comparison to the results using the network model and the assumptions made for restoration of nodes, it is observed that only with mitigation options in place after the evacuation, a restoration to normal operation is possible even in this very simple example. The IPS will allow for a more complex infrastructure to quantitatively describe the impact taking into account resilience principals.

## Conclusion

Small changes in one place, to one object, can proliferate and spread throughout connected networks causing devastating and seemingly unpredictable large changes elsewhere. Critical infrastructure is fundamental to protect and yet is quite vulnerable to these kinds of effects due to its complexity and interconnectedness, and to the fact that it is often sought after as a target for attacks because of its potentially fatal repercussions. Therefore, having meaningful and accurate modeling of how particular threats and risks can propagate through these critical infrastructure systems would greatly enhance the stakeholders’ ability to better determine where countermeasures would be most effective in limiting the effects, should such an attack occur.

The described SATIE toolkit collects live data from airport systems, which is then analysed by threat prevention and detection systems, which can then elicit an event or even an alarm when a potential vulnerability is being exploited. The centralized user interface allows the Airport Operation Center personnel to have an easy overview of the situation, not requiring inquiring with multiple systems and having to manually determine if suspicious activity is occurring. The two described threat prevention and detection tools address two different subsets of threats: the BIA assess cyber-threats and how they affect the performance of business processes, while the ISP analyses both physical and cyber-threats and how they impact assets negatively. The combination of the two ensures the full coverage of assets and processes within an organization, to both physical and cyber-threats.

Through the test cases of an attack on the PA system, demonstrating not only how the cyber-attack can turn into a physical attack to people gathered outside of the airport, also revealed how it can influence other systems and processes of the organization (i.e. the check-in counters and security check desks). A hybrid approach consisting of a network representation of the airport and an ABM was employed to simulate the processes during the attack on the involved airport systems. The simulations presented here are a first step in the development of the hybrid model for the IPS in SATIE. The network model represents the top-down view based on assumptions about connectivity of nodes and repair times whereas the ABM enables to observe what will happen with only passenger attributes being defined, representing the bottom-up perspective. Further work involves that the network model and ABM need to be aligned in time and the communication between the tools needs to be automated. The ABM specifically is still lacking some parts of human behaviour such as e.g.. group- and panic dynamics. The network model in particular needs to be extended with further airport systems and more mitigation options need to be defined. Finally, the model will be designed to be applicable to other critical infrastructures and the respective impact analysis.

Complex systems are ever-more present in our world as systems and people become even-more connected. When the functioning of these systems is instrumental in upholding and supplying our society with its necessities (e.g. water, electricity, food, movement, etc.), then a more holistic and complex threat modelling is necessary to better predict, understand, and then mitigate its effects. The toolkit used in SATIE aims to clear the sky for future endeavours of improving cyber- and physical security around the world.
